# Mechanochemical synthesis of alumina-based catalysts enriched with vanadia and lanthana for selective catalytic reduction of nitrogen oxides

**DOI:** 10.1038/s41598-022-25869-w

**Published:** 2022-12-09

**Authors:** Ewelina Weidner, Rabindra Dubadi, Bogdan Samojeden, Adam Piasecki, Teofil Jesionowski, Mietek Jaroniec, Filip Ciesielczyk

**Affiliations:** 1 Institute of Chemical Technology and Engineering, Faculty of Chemical Technology, Poznan University of Technology, Berdychowo 4, 60965 Poznan, Poland; 2 Department of Chemistry and Biochemistry, Kent State University, Kent, OH 44242 USA; 3 Department of Fuel Technology, Faculty of Energy and Fuels, AGH—University of Science and Technology, Al. A. Mickiewicza 30, 30059 Krakow, Poland; 4 Institute of Materials Engineering, Faculty of Materials Engineering and Technical Physics, Poznan University of Technology, Jana Pawła II 24, 60965 Poznan, Poland

**Keywords:** Catalyst synthesis, Catalytic mechanisms, Heterogeneous catalysis, Catalyst synthesis, Materials for energy and catalysis, Environmental chemistry

## Abstract

Novel alumina-based materials enriched with vanadia and lanthana were successfully synthesized via in situ modification using a mechanochemical method, and were applied in ammonia-induced selective catalytic reduction of nitrogen oxides (SCR process). The synthesis was optimized in terms of the ball milling time (3 or 5 h), vanadium content (0.5, 1 or 2 wt% in the final product), and lanthanum content (0.5 or 1 wt% in the final product). Vanadium (V) oxide was immobilized on an alumina support to provide catalytic activity, while lanthana was introduced to increase the affinity of nitrogen oxides and create more active adsorption sites. Mechanochemical synthesis successfully produced mesoporous materials with a large specific surface area of 279–337 m^2^/g and a wide electrokinetic potential range from 60 to (− 40) mV. Catalytic tests showed that the incorporation of vanadia resulted in a very large improvement in catalytic performance compared with pristine alumina, increasing its efficiency from 14 to 63% at 400 °C. The best SCR performance, a 75% nitrogen oxide conversion rate at a temperature of 450 °C, was obtained for alumina enriched with 2 and 0.5 wt% of vanadium and lanthanum, respectively, which may be considered as a promising result.

## Introduction

The high level of pollution with nitrogen oxides has an undeniable negative impact on the environment and public health, which leads to tightening of the regulations on their emission^1–3^. Nitrogen oxides, or NO_x_ (for example NO, NO_2_, N_2_O), are emitted from stationary sources (such as thermal power stations) and from mobile sources (such as vehicle exhausts), making a significant contribution to acid rain, photochemical smog, and ozone layer depletion^4^. Selective catalytic reduction (SCR) is a promising process for decreasing NO_x_ pollution levels. Currently, SCR is widely used in power station boilers, furnaces, and other industrial coal-fired equipment, being the best commercial technology—in terms of efficiency^5^, selectivity, and economics—to control NO_x_ emissions from stationary sources^6^. Moreover, selective catalytic reduction of NO_x_ with NH_3_ (NH_3_-SCR) is reported to be one of the most effective technologies for the removal of NO_x_ from diesel engines^7^. Over the years, various catalysts have been used in SCR-NO_x_. These materials can be divided into three main groups: (i) V-based oxide catalysts, (ii) Cu or Fe zeolite catalysts, and (iii) vanadium-free oxide catalysts. Due to their high NO_x_ reduction activity, oxide catalysts containing vanadium, particularly commercial V_2_O_5_-WO_3_/TiO_2_ and V_2_O_5_-MoO_3_/TiO_2_, are most often used^8–10^. However, they exhibit certain drawbacks, including low resistance to SO_2_ and H_2_O poisoning, and a narrow operating temperature range (300–400 °C) in the case of NH_3_-SCR^11^. It is widely known that vanadium-containing catalysts supported on alumina, at temperatures approaching 400 °C and at low SO_2_ concentrations (as found in many flue gases), exhibit high resistance to deactivation by SO_2_ poisoning^12–14^. Due to the presence of sulfur compounds (mainly sulfur oxides, SO_x_) in all gas streams containing NO_x_, this feature is extremely important in air pollution control^11^. In addition, the use of a highly mesoporous support may result in increased surface area, which can lead to a higher number of active sites, enabling greater dispersion of vanadia and improving overall catalytic activity. Miyamoto et al. claimed that ammonia is strongly adsorbed adjacent to V = O sites as NH_4_^+^, and that the reaction rate is directly proportional to the number of surface V = O bonds^15^. To enhance NH_3_ adsorption capacity the Al_2_O_3_–vanadia material reported in this study was modified by introducing lanthana during the synthesis. SCR catalysts have previously been modified with La compounds. In the case of formic acid decomposition under SCR-relevant conditions, the addition of a small amount of lanthana to the catalyst led to a base-induced promotional effect^16^. The promotional effect of a basic gas phase reactant (ammonia) on formic acid decomposition activity was achieved catalytically^17,18^. Moreover, lanthana has been reported to cause substantial improvement in the adsorption capability of various materials^19^, which may be beneficial in the case of NH_3_–SCR. This phenomenon is due to the electron configuration in La, which means that its ions can react with functional groups of Lewis acids^20^.

Co-precipitation and sol–gel synthesis are the most popular methods for obtaining oxide systems for use as catalysts in the process of selective reduction of nitrogen oxides^21–23^. It is desirable to obtain more environmentally friendly catalysts using a solvent-free synthesis method. Mechanochemical synthesis of advanced materials is currently propagated as a favorable alternative to traditional solution-based methods, which involve heating, addition of expensive or hazardous reagents, and multi-step processing^24^. In conventional chemical synthesis the solvent often plays a key role in energy dispersion, dissolution/solvation, and the transportation of chemicals. An efficient mixing process can overcome the problem of high solvent consumption by enabling solid phase reactions using only nominal amounts of solvent (wet ball milling or liquid-assisted grinding). This approach enables chemical transformations to be induced by mechanical means such as compression, shearing, or friction^25^. In terms of physicochemical properties, mechanochemical synthesis can afford materials with higher surface area and surface energy by altering their structure, chemical composition and/or chemical reactivity throughout the milling process^26^. These parameters are of particular importance in the design and preparation of advanced materials for a range of catalytic applications. There are many reports on the use of mechanochemical protocols to obtain various catalysts (supported metal nanoparticles, nanocomposites, and nanomaterials) with improved catalytic activity and selectivity^24,26,27^, demonstrating the potential of this method to provide more sustainable routes for the preparation of catalysts^28^.

Here we report the application of alumina/vanadia/lanthana hybrids, obtained via mechanochemical synthesis, as catalysts in the selective catalytic reduction of nitrogen oxides with ammonia. The main objectives of the study included: (i) optimization of the mechanochemical soft-templating synthesis of Al_2_O_3_/V_2_O_5_/La_2_O_3_; (ii) detailed physicochemical and structural analysis of the resulting catalysts; and (iii) practical application of these catalysts in the selective catalytic reduction of NO_x_. This study is based on the hypothesis that the incorporation of vanadia and lanthana may have a significant impact on the physicochemical and structural properties of the alumina-based materials, as well as on their catalytic performance. It is expected that the combination of alumina as a support, vanadia as an active mass, and lanthana as an activator and element affecting the affinity of the catalyst to the gas phase components should result in the formation of functional materials dedicated for environmental catalysis processes.

## Experimental

### Al_2_O_3_/V_2_O_5_/La_2_O_3_ synthesis

**Figure 1:**
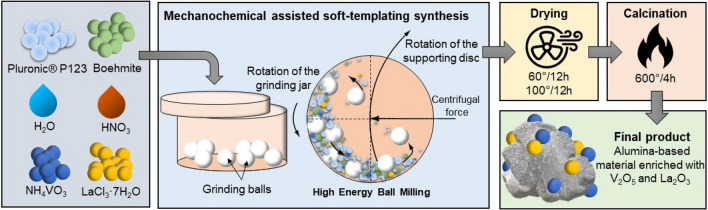
Schematic illustration of the applied synthesis route.

Mesoporous oxide materials, based on alumina modified with vanadium species, were obtained by a one-step mechanochemically assisted soft-templating synthesis, similar to that described elsewhere^29^, as shown in Fig. 1.

Approximately 3 g of (EO)_20_ (PO)_70_(EO)_20_ triblock copolymer (Pluronic^®^ P-123 from BASF, Co), 1.2 g of alumina precursor (boehmite), an appropriate amount of vanadia precursor (NH_4_VO_3_, ≥ 99.0%, from Fisher Scientific Co.), 5 mL of deionized (DI) water, and 100 μL of HNO_3_ (Acros Organics) were introduced to a grinding jar equipped with 8 yttria-stabilized zirconia grinding balls, each of diameter 1 cm. For blank samples, vanadia precursor was not added. In the case of lanthana-modified samples, a specified amount of lanthanum chloride heptahydrate (≥ 99.99%, from Acros Organics) was introduced. The resulting mixture was milled for a set time (3 or 5 h), with a rotation speed of 500 rpm, in a Planetary PM200 mill (Retsch) to pulverize the boehmite particles along with the added metal salts. After milling, the samples were dried at 60 °C for 12 h and then at 100 °C for a further 12 h to evaporate the solvent. The resulting paste-like materials were then calcined in air at 600 °C for 4 h in a tubular quartz furnace to remove the polymeric template and to achieve the desired crystallinity. Next, the samples were cooled naturally to room temperature, and without further purification were subjected to physicochemical analysis. The list of prepared samples is presented in Table 1.

**Table 1:** List of prepared samples.

No	Sample name	Vanadium content (wt %)	Lanthanum content (wt %)	Ball milling time (hours)
1	A-3	–	–	3
2	A-3V0.5	0.5	–	
3	A-3V1	1.0	–	
4	A-3V2	2.0	–	
5	A-5	–	–	5
6	A-5V0.5	0.5	–	
7	A-5 V1	1.0	–	
8	A-5 V2	2.0	–	
9	A-3V2La0.5	2.0	0.5	3
10	A-3V2La1	2.0	1.0	

### Measurements and characterization

X-ray fluorescence (XRF) analysis (Epsilon 4, Malvern Instruments Ltd., UK) and energy-dispersive X-ray spectroscopy (EDX; PTG Prism Si (Li), Princeton Gamma Tech., USA) were used to determine the elemental composition of the obtained materials. Porous structure parameters—BET surface area (*S*_*BET*_), pore width (*w*) at the maximum of the pore size distribution, and total pore volume (*V*_*t*_)—were determined from low-temperature (− 196 °C) nitrogen adsorption/desorption isotherms (ASAP 2020, Micromeritics Instrument Co., USA), by the method reported elsewhere^30^. The pore size distribution (PSD) was calculated using the Kruk–Jaroniec–Sayari method calibrated for cylindrical pores and considering the maximum of the curve as the pore width (*w*)^30,31^. To determine the particle size distribution and particle aggregation, the non-invasive back scattering (NIBS) method was applied, using a Zetasizer Nano ZS (Malvern Instruments Ltd. UK). Scanning electron microscopy (SEM) images were taken using a MIRA3 scanning electron microscope (Tescan, Czech Republic). Powder X-ray diffraction (XRD) measurements were performed on an Empyrean diffractometer (PANalytical, UK) to determine the crystalline structure of the catalyst. Electrokinetic (zeta) potential was determined by the electrophoretic light scattering (ELS) method (Zetasizer Nano ZS, Malvern Instruments Ltd., USA, equipped with an MPT-2 auto titration device).

### Catalytic tests

The performance of selected samples was examined in the selective catalytic reduction of nitrogen oxides (NO_x_) induced with ammonia. The catalytic process was carried out in a fixed-bed flow microreactor under atmospheric pressure at temperatures from 150 to 450 °C, with 200 mg of catalyst. In a standard run, the reaction mixture (800 ppm of NO, 800 ppm of NH_3_, in He with 3% (v/v) addition of O_2_) was introduced into the microreactor through mass flow controllers, maintaining a total flow rate of 100 cm^3^ min^−1^. The catalytic unit downstream of the reactor was used to decompose any NO_2_ that might be formed to NO. The concentrations of NO and N_2_O (a by-product of the reaction) in the product stream were measured every 65 s using an NDIR (nondispersive infrared) sensor from Hartmann and Braun. NO conversion was calculated according to the following formula:

1 $$NO_{conversion} \, = \,\left( {NO_{in} \,{-}\,NO_{out} } \right)\,/\,NO_{in} ,$$

where *NO*_*in*_ is the inlet concentration of NO and *NO*_*out*_ is the outlet concentration of NO.

## Results and discussion

### Synthesis optimization

The focus of this study was the optimization of the synthesis of vanadia- and lanthana-modified alumina and investigation of its catalytic performance in the process of selective catalytic reduction of nitrogen oxides. The first step was to determine the optimal amount of vanadia and grinding time. Increasing the grinding time from 3 to 5 h did not significantly improve the structural properties of the obtained materials, and in some cases the opposite effect was observed (see Table 2). Therefore, on economic grounds, the optimal process time was taken as 3 h. The optimum vanadium content (2 wt%) was determined by gradually increasing the quantity and checking whether the properties of the material were significantly changed. Ultimately, the A-3V2 sample with grinding time 3 h and the highest vanadium content was identified as optimal and was used for further investigations. The conditions for obtaining this sample were further modified by introducing La precursor in quantities equivalent to 0.5 and 1 wt% of La.

**Table 2:** Structural parameters and chemical composition of the analyzed samples.

No	Sample	*S*_*BET*_ (m^2^/g)	*V*_*t*_ (cm^3^/g)	*w* (nm)	Oxide content (%)			Element content (%)			
					Al_2_O_3_	V_2_O_5_	La_2_O_3_	Al	O	V	La
1	A-3	319	1.06	19	100	–	–	51.1	48.9	–	–
2	A-3V0.5	309	1.24	24	97.8	2.2	–	49.7	49.2	1.1	–
3	A-3V1	330	1.39	30	95.6	4.4	–	48.9	48.6	2.5	–
4	A-3V2	317	1.32	31	91.4	8.6	–	49.5	46.3	4.3	–
5	A-5	289	1.26	18	100	–	–	No data			
6	A-5V0.5	337	1.28	18	97.9	2.1	–				
7	A-5 V1	279	1.12	25	96.1	3.9	–				
8	A-5 V2	330	1.31	33	91.5	8.5	–				
9	A-3V2La0.5	286	0.91	29	90.3	8.6	1.1	52.0	43.7	3.7	0.5
10	A-3V2La1	317	1.05	33	89.3	8.7	2.0	50.2	44.7	3.9	1.1

### Morphology of selected samples

**Figure 2:**
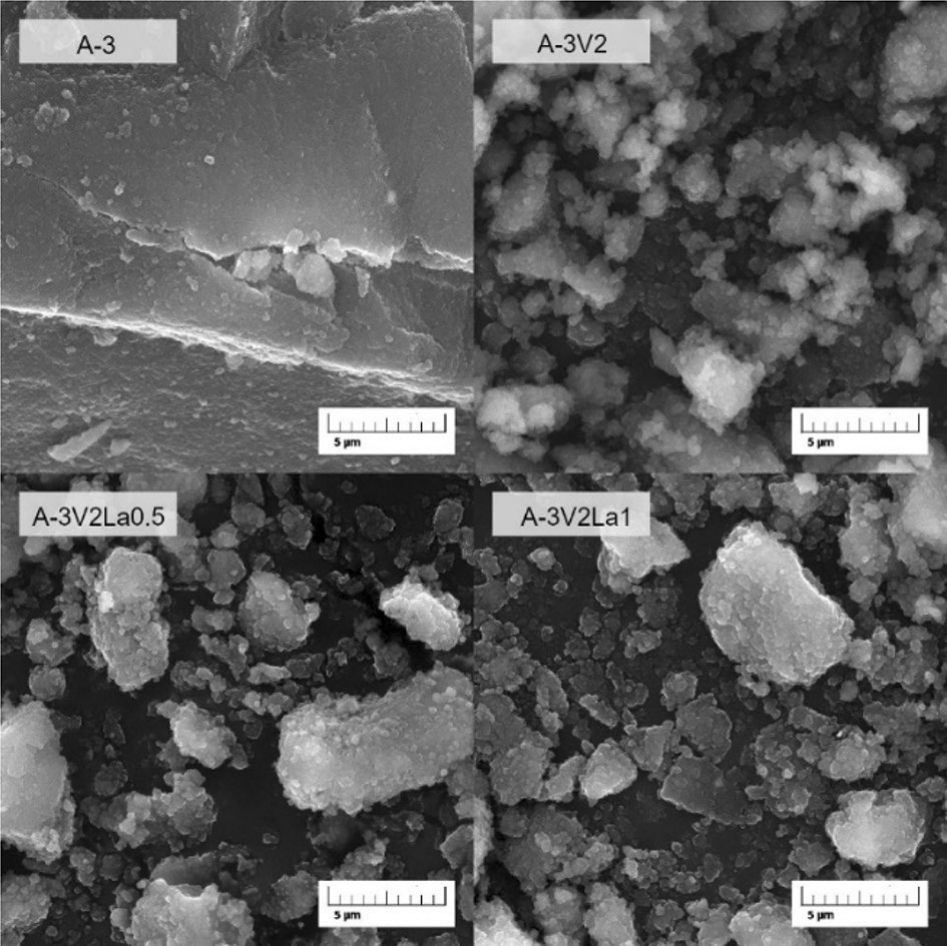
SEM images with 5 µm scale bar for the samples selected for catalytic testing: A-3, A-3V2, A-3V2La0.5 and A-3V2La1.

Scanning electron microscopy (SEM) was used to determine the morphology, shape, and size of individual grains of the obtained materials. The SEM images are presented in Fig. 2. In the case of sample A-3, some large agglomerates are visible. The SEM image also reveals the relatively smooth surface of the particles. The addition of vanadia precursor in sample A-3V2 caused a significant reduction in the size of agglomerates. An increase in the roughness of the material surface can also be observed. In both cases in which Al_2_O_3_ was modified with vanadia and lanthana (A-3V2La0.5 and A-3V2La1), the particles consist of grains having different sizes and irregular shapes, which tend to agglomerate. It can be concluded that the introduction of additional elements to Al_2_O_3_ increases the heterogeneity of its structure.

### Chemical composition of prepared materials

**Figure 3:**
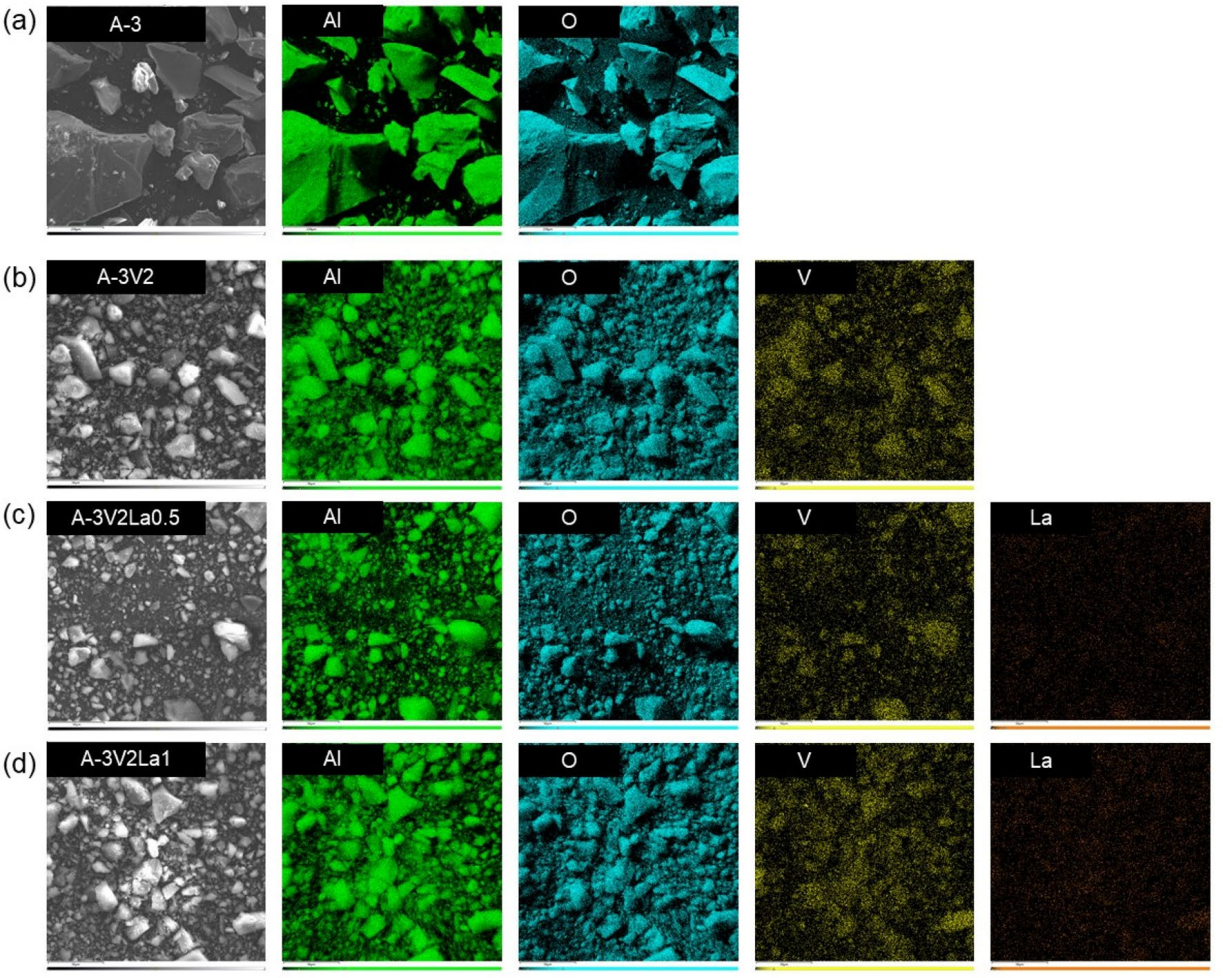
EDX surface mapping of (**a**) A-3, (**b**) A-3V2, (**c**) A-3V2La0.5 and (**d**) A-3V2La1 samples, including SEM image and images showing distribution of Al, O, V and La.

Determination of the chemical composition of the samples enabled indirect confirmation of the effectiveness of the mechanochemical synthesis of materials based on aluminum, vanadium, and lanthanum oxides. Detailed oxide compositions of all synthesized samples are given in Table 2, and EDX surface mappings of samples A-3, A-3V2, A-3V2La0.5 and A-3V2La1 are shown in Fig. 3. The surface mapping images reveal the uniform distribution of all elements on the samples’ surfaces. Furthermore, distinct signals from both doping elements are clearly visible, which provides indirect confirmation of the effectiveness of the synthesis process.

An increase in the percentage of vanadium leads to more intense vanadium signals, confirming the higher V_2_O_5_ content in the samples, as presented in Table 2. What is more, an increase in the content of vanadium, consistent with the assumptions of the synthesis process (see Table 1), confirms the effectiveness of the proposed modification method.

**Figure 4:**
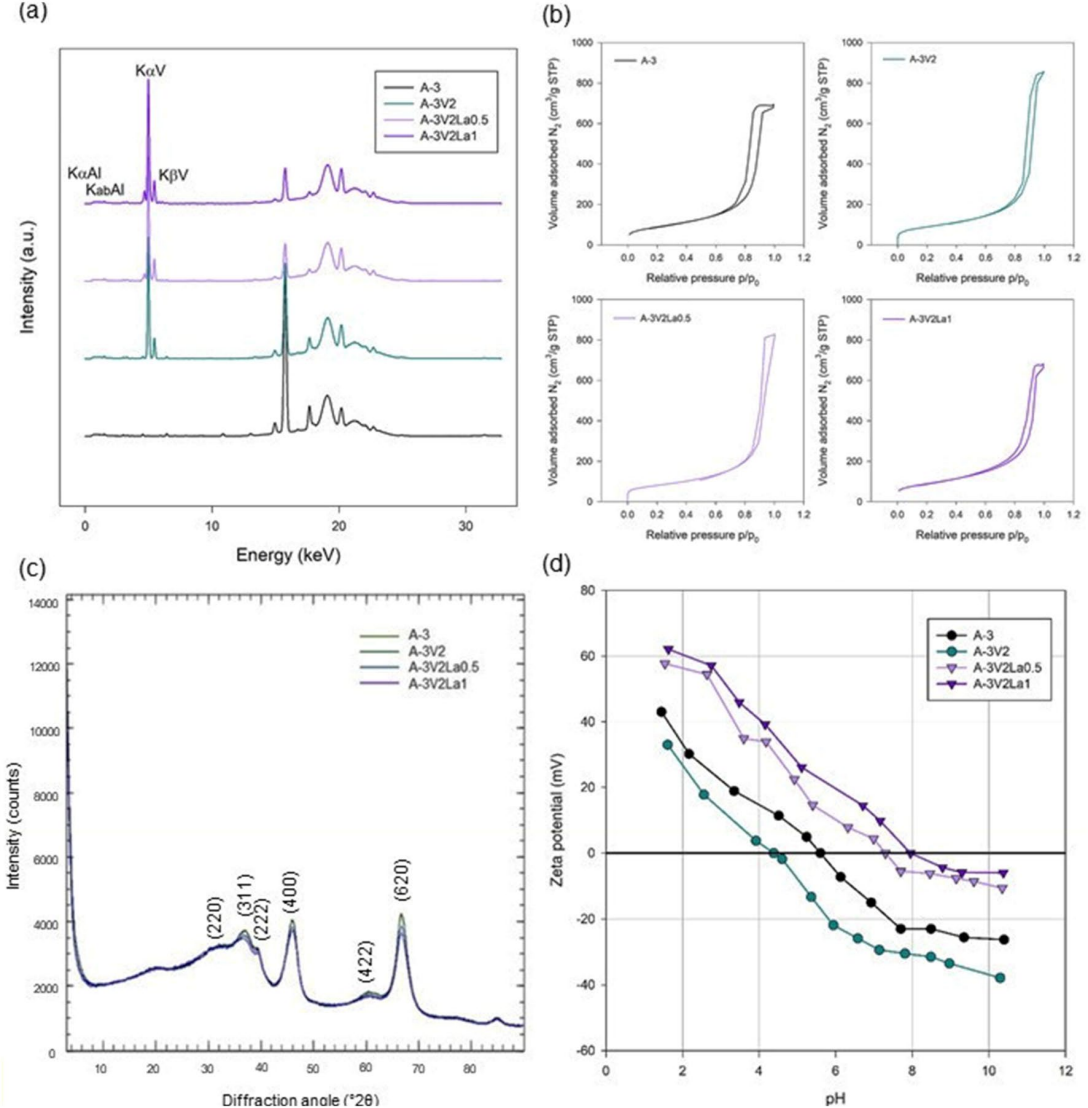
XRF spectra (**a**), adsorption–desorption isotherms (**b**), XRD patterns (**c**), and graphs of zeta potential vs. pH (**d**) obtained for samples A-3, A-3V2, A-3V2La0.5 and A-3V2La1.

The XRF spectra obtained for samples A-3, A-3V2, A-3V2La0.5 and A-3V2La1 are shown in Fig. 4a. For all samples, signals originating from aluminum were detected at around 1.486 keV (KαAl). In samples synthesized with ammonium metavanadate, peaks characteristic of vanadium were observed at around 4.949 keV (KαV) and 5.426 keV (KβV); these reflect the vanadium content, which ranges from 1.1 to 4.3 wt%. In the case of samples prepared with the addition of LaCl_3_, the presence of La, in the form of La_2_O_3_, was also confirmed, with contents of 0.5 and 1.1 wt% for A-3V2La0.5 and A-3V2La1, respectively. These results provide indirect confirmation of both, the effective synthesis of aluminum oxide with incorporated vanadia and lanthana, and the assumed composition of the materials.

### Porous structure parameters

Low-temperature nitrogen adsorption analysis was used to determine the porous properties of the synthesized materials: surface area (*S*_*BET*_), total pore volume (*V*_*t*_), and mesopore size (*w*) at the maximum of the pore size distributions. Data for all samples are given in Table 2. All samples have a relatively large specific surface area, close to 300 m^2^/g; the lowest value (279 m^2^/g) was obtained for sample A-5 V1, and the highest (337 m^2^/g) for sample A-5V0.5. The total pore volume was not significantly affected by the changes in material’s composition. The data also show that increasing the process time from 3 to 5 h did not lead to a significant increase in the surface area of the resulting materials. For this reason, sample A-3V2 was selected as a base material for modification with lanthana. That modification did not significantly affect the surface area of the material. The nitrogen adsorption–desorption curves for samples A-3, A-3V2, A-3V2La0.5 and A-3V2La1 are presented in Fig. 4b. In all curves, the presence of a hysteresis loop is visible. These curves can be characterized as type IV isotherms according to the IUPAC classification, characteristic for mesoporous materials^32^. Isotherms of this type, in combination with an increase in adsorption at high pressures approaching the saturation vapor pressure, are mainly observed for hierarchical porous materials with a wide range of pore size distribution, including meso- and macropores^33^. Moreover, it is known that type IV isotherms represent mono- and multilayer adsorption at low and moderate relative pressures followed by capillary condensation at higher relative pressures^34^. This leads to the conclusion that mechanochemical synthesis is an effective method for obtaining mesoporous alumina/vanadia/lanthana materials.

### Crystalline structure of the obtained materials

The XRD patterns of the obtained powders are presented in Fig. 4c. The patterns for all samples contain diffraction peaks corresponding to signals originating from Al_2_O_3_ with cubic crystal symmetry exhibiting a face-centered lattice. Signals originating from α-Al_2_O_3_ (peaks at 400, 422 and 620), γ-Al_2_O_3_ (peaks at 220, 311, 222 and 140) and θ-Al_2_O_3_ (peak at 140) are visible in the patterns. All diffraction peaks in the patterns suggest that they are consistent with the standard Al_2_O_3_ pattern (JCPDS database, Card Number 79-1558)^35–37^. The addition of V or La had no significant impact on the course of the curve, and thus on the crystalline structure of the sample. No diffraction peaks from any other chemical species are detectable in the diffraction patterns.

### Surface charge properties

The materials surface electrokinetic potential may be important for assessing the effectiveness of adsorption processes, being a determining factor for catalytic degradation efficiency. The value of the surface charge of the material controls its bonding with the adsorbate via the mechanism of electrostatic attraction. Figure 4d shows the electrokinetic potential as a function of the pH of the solution for the four selected materials.

For all tested materials, the curves follow the same trend—the materials have positive zeta potential at slightly acidic pH, reaching the isoelectric point (IEP) around a neutral pH of 6–8, and developing a negative charge in a more basic environment. The zeta potential values decrease with increasing pH due to the smaller number of H^+^ ions. In more alkaline environment, due to the presence of hydroxyl ions (OH^−^), the negative charge builds up at the sample surface^38^. Consequently, an increase in pH may lead to a reduction in the zeta potential value. For the pristine Al_2_O_3_ material (A-3) the maximum zeta potential is 43 mV and the minimum is − 21 mV. The addition of vanadia precursor during the synthesis caused a reduction in the material’s potential range, to 33–(− 39) eV. Similar changes in the potential have been observed in the case of other inorganic oxides doped with vanadium^39^. While the addition of vanadia precursor reduces this range, the incorporation of lanthana results in a significant increase in the pH range in which the zeta potential remains positive. The presence of a positive charge on the material’s surface is probably related to the protonization of OH groups.

The isoelectric point (IEP), evaluated from the graph of zeta potential vs. pH, plays an important role in the process of heterogeneous catalysis, because it affects the adsorption capacity of reactants on the catalyst surface^40^. The IEPs of A-3, A-3V2, A-3V2La0.5 and A-3V2La1 were found to lie at pH values of 5.6, 4.4, 7.3 and 7.9, respectively. This means that the materials obtained are positively charged in solutions with pH values lower than these points, and negatively charged in solutions with pH above these points.

### Catalytic activity

**Figure 5:**
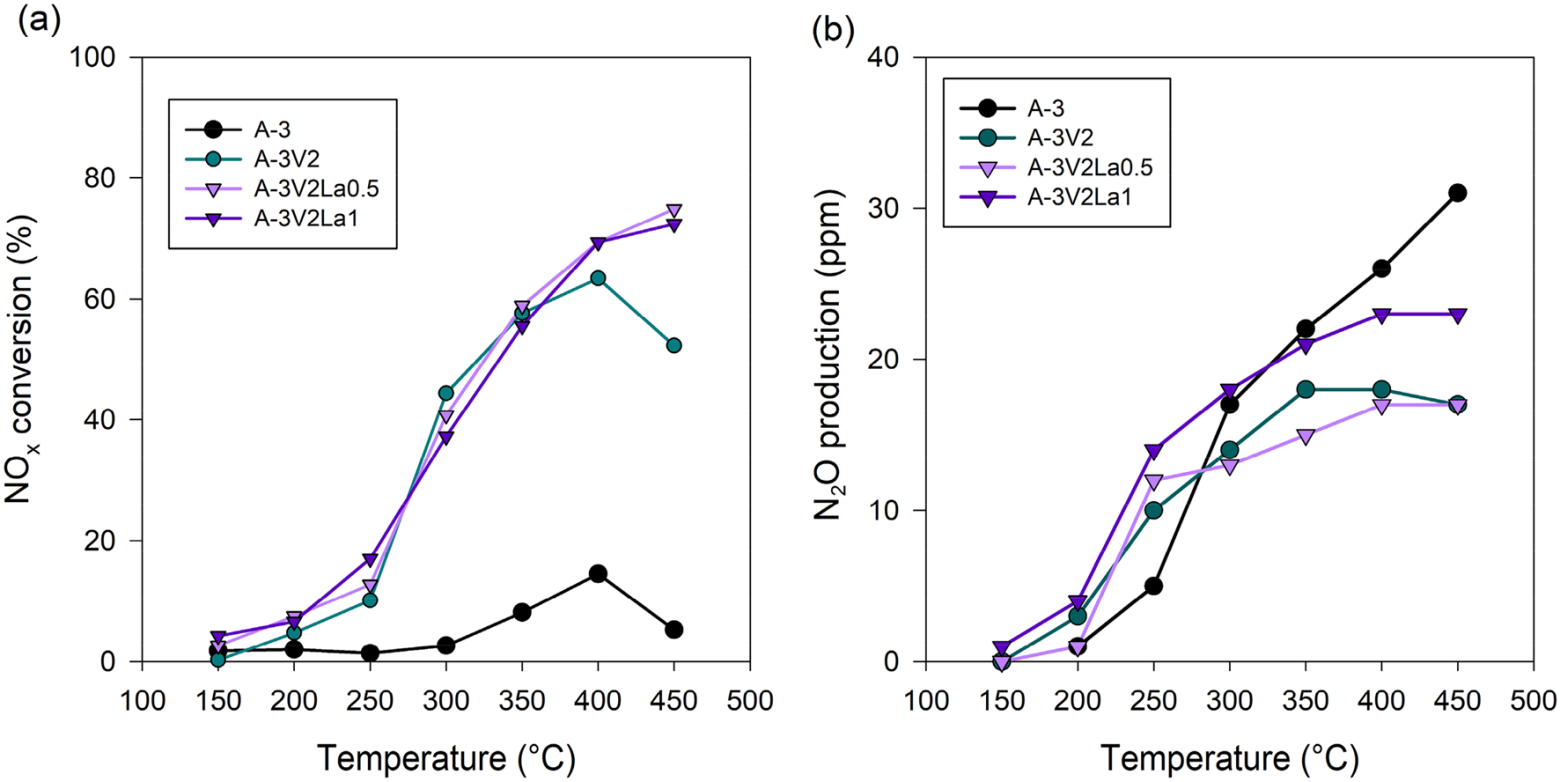
Results of catalytic tests using the selected materials, including NO_x_ conversion (**a**) and by-product (N_2_O) production (**b**).

The catalytic abilities of selected aluminum oxide-based materials were tested in the process of ammonia-induced selective catalytic reduction of NO_x_, within the temperature range 150–450 °C. The test results are presented in Fig. 5.

**Figure 6:**
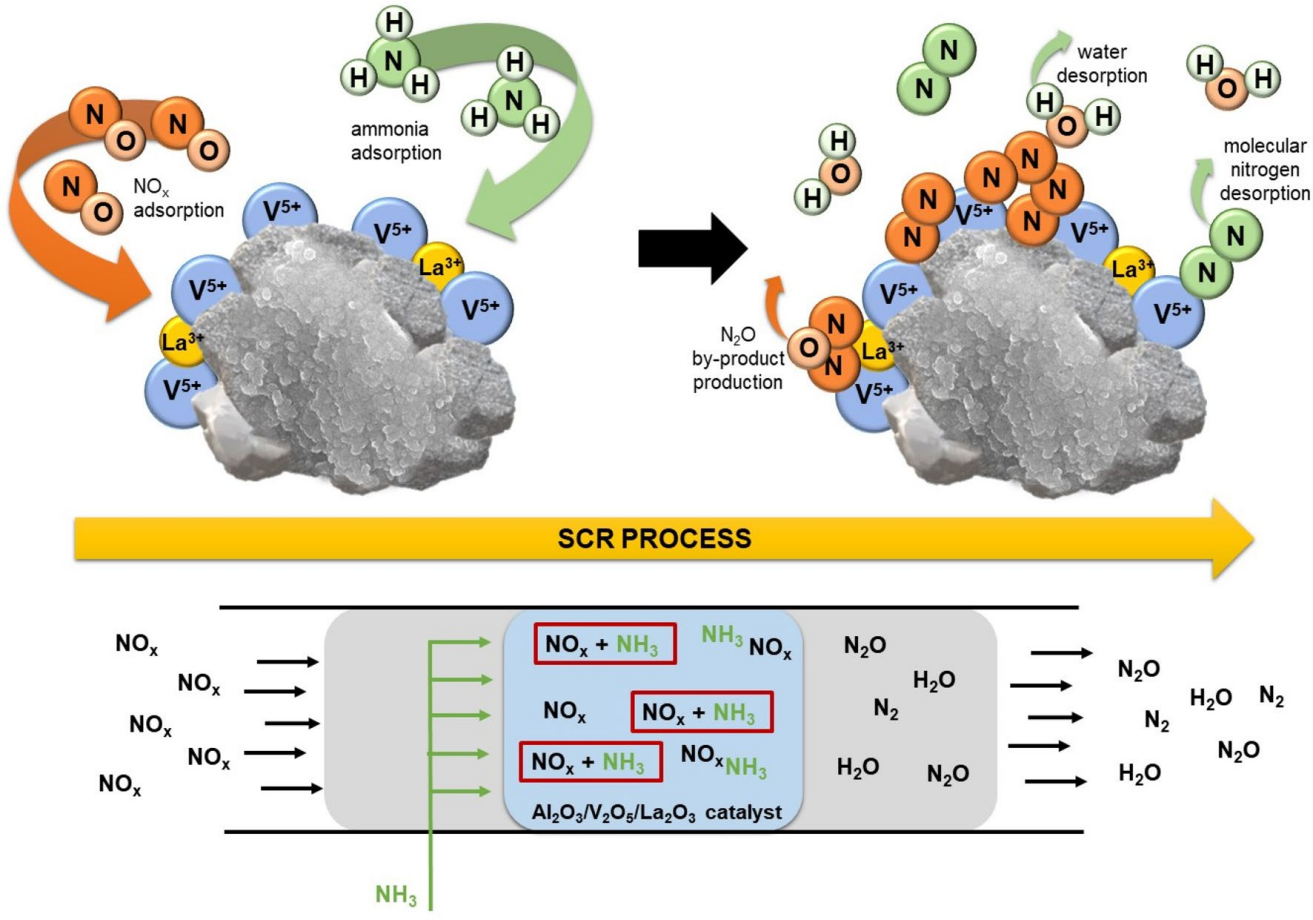
Proposed mechanism of selective catalytic reduction of NO_x_ induced with ammonia on Al_2_O_3_/V_2_O_5_/La_2_O_3_ catalyst.

Generally, as the reaction temperature increases, an upward trend in the reduction of nitrogen oxides is observed, as expected. The lowest catalytic activity is observed for pure Al_2_O_3_ (A-3), which achieves a maximum reduction of 14% at 400 °C. For this material, the beginning of NO_x_ production is observed above 400 °C. A very large improvement in catalytic activity is observed for the vanadium-containing sample A-3V2 in comparison with the pristine material. The highest NO_x_ reduction rate of 63% was obtained with this material at 400 °C, and was significantly higher than the result for the pure Al_2_O_3_ material. This is probably related to the fact that the enrichment of alumina with vanadia significantly improves its catalytic properties. As mentioned in the introduction, ammonia is strongly adsorbed adjacent to V = O sites as NH_4_^+^, and the reaction rate is directly proportional to the number of surface V = O bonds^15^, which facilitate the SCR reaction. Moreover, the presence of nitrogen in the form of NH_4_^+^ may significantly strengthen the catalytic activity of the materials. Unfortunately, for sample A-3V2, the conversion of NO_x_ at temperatures above 400 °C was found to be problematic. However, for the samples modified with lanthana this problem disappeared. The incorporation of lanthana into the structure of the Al_2_O_3_/V_2_O_5_ materials did not significantly improve their catalytic performance in the temperature range 150–400 °C, but enabled elimination of the production of nitrogen oxides at temperatures above this range. This behavior may also be due to the wider range of positive surface charge of lanthana-modified samples. By-product production was constantly monitored during the experiments, and the results are presented in Fig. 5b. For all samples, the amount of by-product increased gradually with increasing reaction temperature. However, in the case of vanadia- and lanthana-modified samples the amount of N_2_O did not exceed 23 ppm in the whole temperature range. For the pure Al_2_O_3_ sample higher N_2_O production was observed, reaching almost 31 ppm at 450 °C, which is nevertheless fairly low. Based on the results, a mechanism for the catalytic action of Al_2_O_3_/V_2_O_5_/La_2_O_3_ materials was proposed (see Fig. 6).

Nitrogen oxides are adsorbed on the surface of Al_2_O_3_/V_2_O_5_/La_2_O_3_ and, due to the presence of active vanadium sites, ammonia reacts with them. Moreover, the incorporation of lanthana causes an increase in the number of active sites on the catalyst’s surface, which facilitates the adsorption of nitrogen oxides and thus makes their selective catalytic reduction more effective. Ammonia holds a dominant position in competitive adsorption between NH_3_ and NO on similar vanadium-containing metal oxide catalysts, and so analogous behavior probably occurs in the case of Al_2_O_3_/V_2_O_5_/La_2_O_3_. Therefore, the bonds between nitrogen and oxygen are broken, enabling the formation of molecular nitrogen, water, and N_2_O. Nitrogen oxides can be adsorbed on the surface of the catalyst and then transformed into monodentate species of nitrite and nitrates, which is favored by the presence of O_2_ and significantly inhibits NH_3_ adsorption, hindering the SCR reaction^41^.

### Comparison with previous studies

Alumina-based materials modified with various metal species have previously been used as catalysts in SCR processes. However, they have mostly been investigated in hydrocarbon-assisted SCR, whereas NH_3_-SCR is currently the leading technology for the elimination of nitrogen oxides from diesel engines. Oton et al.^11^ modified alumina with Pt, Co, Fe and Ni by a wet impregnation procedure, and tested them in the selective catalytic reduction of NO_x_ by CO. Platinum- and cobalt-containing alumina achieved an NO_x_ conversion rate of almost 100% at 400 °C, while Fe–Al_2_O_3_ achieved around 60% and Ni–Al_2_O_3_ 0%. By comparison, the vanadia-modified alumina sample obtained in this work reduced 63% of nitrogen oxides at that temperature. Kumar et al.^42^ prepared a series of La_2_O_3_-modified Al_2_O_3_ supports for Ag, using a wet impregnation method, and obtained materials with relatively high surface areas (161–281 m^2^/g). These materials produced high N_2_O conversion rates, reaching 100% on 5%Ag/1%La_2_O_3_–Al_2_O_3_ at 600 °C. However, at lower temperatures these materials gave much worse results—less than 40% reduction for all samples—while in our study the highest NO_x_ reduction rate of 75% was obtained for the A-3V2La0.5 material at 450 °C. Nascimento et al.^43^ investigated Al_2_O_3_-La_2_O_3_-based catalysts modified with bimetallic species of Ni-Mo, Co-Mo and Pt–Mo by a wet impregnation method, and investigated their performance in a CO-assisted SCR process. At 400 °C, Ni–Mo/Al_2_O_3_–La_2_O_3_, Co–Mo/Al_2_O_3_–La_2_O_3_, and Pt–Mo/Al_2_O_3_–La_2_O_3_ achieved NO_x_ conversion rates of 0%, < 40% and < 60%, respectively. By contrast, synthesized under presented study A-3V2La0.5 material enabled to achieve a 70% conversion rate at 400 °C, higher than that achieved by the Pt–Mo/Al_2_O_3_–La_2_O_3_ sample, justifying the claim that vanadium compounds are promising modifiers of catalysts for SCR processes.

## Conclusions

In this work, an effective mechanochemical synthesis of alumina-based oxide materials, and their successful enrichment with vanadium and lanthanum species were accomplished via *in situ* modification. The presence of V (1.1–4.3 wt%) and La (0.5–1.1 wt%) in the modified samples was confirmed by EDX analysis. XRF analysis further confirmed the presence of these elements in the oxide forms V_2_O_5_ (2.2–8.8 wt%) and La_2_O_3_ (1.1–2.0 wt%). All nitrogen adsorption–desorption isotherms were classified as type IV, characteristic for mesoporous materials, due to the presence of a hysteresis loop. The proposed synthesis route led to materials with a large specific surface area of 279–337 m^2^/g. While SEM images of pristine Al_2_O_3_ show agglomerates of significant size, all other samples reveal irregular structures with various grain sizes and degrees of agglomeration, which suggests that the introduction of additional metal-containing species to Al_2_O_3_ increases the structural heterogeneity of the material. Surface charge properties were determined by measurements of electrokinetic potential. All of the samples exhibited similar curves, with positive zeta potential values at acidic pH, reaching the isoelectric point around a neutral pH of 6–8, and developing a negative charge in more basic environments. The incorporation of vanadia caused a decrease in the zeta potential, while the addition of lanthana caused its significant increase. It is concluded that the proposed mechanochemical synthesis is an effective method for obtaining mesoporous alumina/vanadia/lanthana hybrids.

Selected samples were subjected to SCR catalytic testing. As expected, pristine alumina exhibited the lowest catalytic activity, achieving a maximum reduction of 14% at 400 °C. The incorporation of vanadia resulted in a very large improvement in catalytic performance, with the efficiency of 63% at 400 °C. The addition of lanthana eliminated the re-production of nitrogen oxides at temperatures above 400 °C. Among the tested samples, A-3V2La0.5 and A-3V2La1 achieved the best SCR catalytic performance, reaching conversion rates of 75% and 71%, respectively, at 450 °C, which may be considered as a promising result. This study has shown that alumina with incorporated vanadia and lanthana exhibits high catalytic performance in the ammonia-assisted SCR reaction, and may also be a beneficial material for other applications.

## Data Availability

All data generated or analyzed during this study are included in this published article.

## References

[1] Shang Z (2016). The study of C_3_H_8_-SCR on Ag/Al_2_O_3_ catalysts with the presence of CO. Catal. Today.

[2] Zhang X, Zhang X (2019). Promotion of surface acidity and surface species of doped Fe and SO_4_^2^^−^ over CeO_2_ catalytic for NH_3_-SCR reaction. Molecular Catalysis.

[3] Zhang C, Sun C, Wu M, Lu K (2019). Optimisation design of SCR mixer for improving deposit performance at low temperatures. Fuel.

[4] Liu S, Wang H, Wei Y, Zhang R, Royer S (2019). Morphology-oriented importance of structural and textural properties ZrO_2_-supported vanadium oxide for the NH_3_-SCR process. ACS Appl. Mater. Interfaces.

[5] Nova I, Ciardelli C, Tronconi E, Chatterjee D, Bandl-Konrad B (2006). NH_3_–NO/NO_2_ chemistry over V-based catalysts and its role in the mechanism of the fast SCR reaction. Catal. Today.

[6] Forzatti P, Lietti L, Tronconi E (2010). Nitrogen oxides removal—Industrial. Encycl. Catal..

[7] Liu K, Liu F, Xie L, Shan W, He H (2015). DRIFTS study of a Ce-W mixed oxide catalyst for the selective catalytic reduction of NO_x_ with NH_3_. Catal. Sci. Technol..

[8] Mrad R, Aissat A, Cousin R, Courcot D, Siffert S (2015). Catalysts for NO_x_ selective catalytic reduction by hydrocarbons (HC-SCR). Appl. Catal. A.

[9] Li X (2018). Interaction of phosphorus with a FeTiO_x_ catalyst for selective catalytic reduction of NO_x_ with NH_3_: Influence on surface acidity and SCR mechanism. Chem. Eng. J..

[10] Wang AD (2018). NH_3_-SCR performance of WO_3_ blanketed CeO_2_ with different morphology: Balance of surface reducibility and acidity. Catal. Today.

[11] Oton LF (2019). Selective catalytic reduction of NO_x_ by CO (CO-SCR) over metal-supported nanoparticles dispersed on porous alumina. Adv. Powder Technol..

[12] Bauerle GL, Wu SC, Nobe K (1978). Parametric and durability studies of NO_x_ reduction with NH_3_ on V_2_O_5_ catalysts. Ind. Eng. Chem. Res..

[13] Bauerle GL, Wu SC, Nobe K (1975). Catalytic reduction of nitric oxide with ammonia on vanadium oxide and iron-chromium oxide. Ind. Eng. Chem. Res..

[14] Nam I-S, Eldridge JW, Kittrell JR (1986). Deactivation of a vanadia-alumina catalyst for NO reduction by NH_3_. Ind. Eng. Chem. Res..

[15] Miyamoto A, Yamazaki Y, Inomata M, Murakami Y (1981). Determination of the number of V=O species on the surface of vanadium oxide catalysts. 1. Unsupported V_2_O_5_ and V_2_O_5_/TiO_2_ treated with an ammoniacal solution. J. Phys. Chem..

[16] Sridhar AM (2018). Mechanistic implications of lanthanum-modification on gold-catalyzed formic acid decomposition under SCR-relevant conditions. Appl. Catal. B Environ..

[17] Sridhar M, van Bokhoven AJ, Kröcher O (2014). Effect of ammonia on the decomposition of ammonium formate over Au/TiO_2_ under oxidizing conditions relevant to SCR: Enhancement of formic acid decomposition rate and CO_2_ production. Appl. Catal. A Gen..

[18] Sridhar M, Ferri D, Elsener M, van Bokhoven JA, Kröcher O (2015). Promotion of ammonium formate and formic acid decomposition over Au/TiO_2_ by support basicity under SCR-relevant conditions. ACS Catal..

[19] Wang C (2019). A simple method to improve the adsorption properties of drinking water treatment residue by lanthanum modification. Chemosphere.

[20] Goscianska J, Ciesielczyk F (2019). Lanthanum enriched aminosilane-grafted mesoporous carbon material for efficient adsorption of tartrazine azo dye. Microporous Mesoporous Mater..

[21] Wan Y, Zhao W, Tang Y, Li L, Wang H, Cui Y, Gu J, Li Y, Shi J (2014). Ni-Mn bi-metal oxide catalysts for the low temperature SCR removal of NO with NH_3_. Appl. Catal. B.

[22] Li W, Liu H, Chen Y (2017). Promotion of transition metal oxides on the NH_3_-SCR performance of ZrO_2_-CeO_2_ catalyst. Front. Environ. Sci. Eng..

[23] Nie J, Wu X, Ma Z, Xu T, Si Z, Chen L, Weng D (2014). Tailored temperature window of MnO_x_-CeO_2_ SCR catalyst by addition of acidic metal oxides. Chin. J. Catal..

[24] Szczęśniak B, Borysiuk S, Choma J, Jaroniec M (2020). Mechanochemical synthesis of highly porous materials. Mater. Horizons.

[25] Jarvis KA, Wang C, Manthiram A, Ferreira PJ (2014). The role of composition in the atomic structure, oxygen loss, and capacity of layered Li–Mn–Ni oxide cathodes. J. Mater. Chem. A.

[26] Xu C, De S, Balu AM, Ojeda M, Luque R (2015). Mechanochemical synthesis of advanced nanomaterials for catalytic applications. Chem. Commun..

[27] Zhao L-Y, Dong X-L, Lu A-H (2020). Mechanochemical synthesis of porous carbons and their applications in catalysis. ChemPlusChem.

[28] Ralphs K, Hardacre C, James SL (2013). Application of heterogeneous catalysts prepared by mechanochemical synthesis. Chem. Soc. Rev..

[29] Szczęśniak B, Choma J, Jaroniec M (2021). Facile mechanochemical synthesis of highly mesoporous γ-Al_2_O_3_ using boehmite. Microporous Mesoporous Mater..

[30] Kruk M, Jaroniec M (2001). Gas adsorption characterization of ordered organic-inorganic nanocomposite materials. Chem. Mater..

[31] Kruk M, Jaroniec M, Sayari A (1997). Application of large pore MCM-41 molecular sieves to improve pore size analysis using nitrogen adsorption measurements. Langmuir.

[32] Cychosz KA, Thommes M (2018). Progress in the physisorption characterization of nanoporous gas storage materials. Engineering.

[33] Kumar KV (2019). Characterization of adsorption site energies and heterogeneous surfaces of porous materials. J. Mater. Chem. A.

[34] Donohue MDU, Aranovich GL (1998). Classification of Gibbs adsorption isotherms. Adv. Colloid Interface Sci..

[35] Chaieb T, Delannoy L, Louis C, Thomas C (2016). Promoting Ag/Al_2_O_3_ performance in low-temperature H_2_–C_3_H_6_-SCR by thermal pretreatment of γ-alumina in water. Catal. Lett..

[36] Ansari SA, Husain Q (2011). Immobilization of Kluyveromyces lactis beta galactosidase on concanavalin A layered aluminium oxide nanoparticles—Its future aspects in biosensor applications. J. Mol. Catal. B. Enzym..

[37] Djebaili K, Mekhalif Z, Boumaza A, Djelloul A (2015). XPS, FTIR, EDX, and XRD analysis of Al_2_O_3_ scales grown on PM2000 alloy. J. Spectrosc..

[38] Huo W (2018). Effect of zeta potential on properties of foamed colloidal suspension. J. Eur. Ceram. Soc..

[39] Weidner E, Kurc B, Samojeden B, Pigłowska M, Kołodziejczak-Radzimska A, Jesionowski T, Ciesielczyk F (2022). Exploiting the multifunctionality of a designed vanadium-doped ZnO hybrid for selective catalytic reduction of NO_x_ and electrochemical applications. J. Environ. Chem. Eng..

[40] Nethaji S, Tamilarasan G, Neehar P, Sivasamy A (2017). Visible light photocatalytic activities of BiOBr-activated carbon (derived from waste polyurethane) composites by hydrothermal process. J. Environ. Chem. Eng..

[41] Zhang Y, Yue X, Huang T, Shen K, Lu B (2018). In situ DRIFTS studies of NH_3_-SCR mechanism over V_2_O_5_-CeO_2_/TiO_2_-ZrO_2_ catalysts for selective catalytic reduction of NO_x_. Materials (Basel).

[42] Kumar A, Venkateshwarlu V, Rao K, Lingaiah N, Prasad P (2013). Alumina supported silver lanthana catalyst for N_2_O decomposition. Int. J. Chem. Phys. Sci..

[43] Nascimento P (2020). Combined promoting effect of molybdenum on the bimetallic Al_2_O_3_-La_2_O_3_ catalysts for NO_x_ reduction by CO. Fuel.

